# New Amphiphilic Imidazolium/Benzimidazolium Calix[4]arene Derivatives: Synthesis, Aggregation Behavior and Decoration of DPPC Vesicles for Suzuki Coupling in Aqueous Media

**DOI:** 10.3390/nano10061143

**Published:** 2020-06-10

**Authors:** Vladimir Burilov, Ramilya Garipova, Elsa Sultanova, Diana Mironova, Ilya Grigoryev, Svetlana Solovieva, Igor Antipin

**Affiliations:** 1 Kazan Federal University, 18 Kremlevskaya st., 420008 Kazan, Russia; aukhadieva.ramilya@yandex.ru (R.G.); elsultanova123@gmail.com (E.S.); mir_din@mail.ru (D.M.); os18sir@yandex.ru (I.G.); iantipin54@yandex.ru (I.A.); 2 A.E.Arbuzov Institute of Organic & Physical Chemistry, 8 Arbuzov str., 420088 Kazan, Russia; evgersol@yandex.ru

**Keywords:** calix[4]arene, NHC complex, Suzuki–Miyaura coupling, DPPC vesicles

## Abstract

In this study, new types of amphiphilic calix[4]arene derivatives bearing *N*-alkyl/aryl imidazolium/benzimidazolium fragments were designed and synthesized by two step transformation: Regioselective Blanc chloromethylation of distal-di-O-butyl calix[4]arene and subsequent interaction with *N*-Substituted imidazole/benzimidazole. Critical aggregation concentration (CAC) values were estimated using pyrene fluorescent probe. Obtained macrocycles were found to form submicron particles with electrokinetic potential +44–+57 mV in aqueous solution. For the first time it was found that amphiphilic calixarene causes the fast transformation of 1,2-dipalmitoyl-sn-glycero-3-phosphocholine (DPPC) multilamellar vesicles into unilamellar ones and leads to the ordering of the lipid in membranes at the molar calixarene/DPPC ratio more than 0.07. In situ complexes of calixarene aggregates with Pd(OAc)_2_ were found to be active in Suzuki–Miyaura coupling of 1-bromo-4-nitrobenzene with phenylboronic acid in water. It was shown that bulky *N*-substituents of heterocycle decrease the catalytic activity of the aggregates. These result can be assigned to the inhibition effect of Pd(II) complex in situ formation by bulky substituents located on the aggregate surface. Embedding of the most active palladium *N*-heterocyclic carbene (NHC) complex with methylimidazolium headgroups into DPPC vesicles enhances its catalytic activity in Suzuki–Miyaura coupling.

## 1. Introduction

Effective approaches to the new carbon–carbon and carbon–heteroatom bond formation are attracting a lot of attention among chemists [1,2]. One of the most important tools is cross-coupling reactions, available to synthetic chemists in their quest to create artificial (or reproduce natural) organic scaffolds [3]. In recent decades, a special place in the cross-coupling catalysis has been occupied by palladium complexes with *N*-heterocyclic carbene (NHC) ligands [4,5,6]. Exponential growth of interest to palladium NHC complexes is quite expected: (i) NHC complexes can be easily synthesized both ex situ and in situ in the reaction medium from palladium salt and appropriate *N*-heterocycles in the presence of base; (ii) the electron-donor and steric properties of the ligand can be easily regulated by substituents on the heterocycle core; (iii) synthesis of precursors (salts of *N*-heterocycles) can be carried out easily through *N*-alkylation of appropriate *N*-heterocycles (imidazole derivatives are used most often). However, the most attractive property of NHC complexes is their stability to moisture and air oxygen, thus allowing to perform specific catalytic reactions in aqueous media without inert atmosphere [7,8]. The application of water as solvent brings down the overall cost and significantly reduces the health risks due to its non-toxicity and nonflammability, unlike most organic solvents [9]. However, not all organic substances are water-soluble and to solve this problem the surfactants are usually used. Thus, NHC complexes with amphiphilic properties allow to combine both micellar and metallocomplex types of catalysis in aqueous media [10,11]. So, the design of new amphiphilic NHC complexes is very promising for enhancing the cross-coupling reactions’ efficiency.

Recently, much attention has been paid to NHC complexes on the calixarene platform [12]. Embedding of NHC units on the calixarene scaffold can serve as a tool to change the environment of central metal due to steric hindrance and electronic effects. A calixarene cavity can also impact on catalytic transformations [13]. The unique structure of calix[4]arenes as well as the variety of stereoisomeric forms and the facile functionalization of both upper and lower rims allows to create amphiphilic structures with spatial separation of lipophilic and hydrophilic domains, that is why they have been widely used to construct macrocyclic amphiphiles [14,15,16,17].

Herein we report the synthesis, aggregation behavior, embedding into DPPC vesicles and catalytic activity of their in situ complexes with Pd(OAc)_2_ in Suzuki–Miyaura coupling of new amphiphilic calix[4]arene derivatives containing two imidazolium/benzimidazolium headgroups and two lipophilic fragments on the upper and lower rim, respectively.

## 2. Materials and Methods

### 2.1. Characterisation Methods

TLC was performed on Merck UV 254 plates with Vilber Lourmat VL-6.LC UV lamp (254 nm) control. Elemental analysis of synthesized compounds was done on the PerkinElmer PE 2400 CHNS/O Elemental Analyzer. NMR spectra were recorded on Bruker Avance 400 Nanobay with signals from residual protons of deuterated solvents (CDCl_3_ or DMSO-d6) as the internal standard. MALDI mass spectra were measured on UltraFlex III TOF/TOF with PNA matrix, laser Nd:YAG, λ = 355 nm. The IR spectra were recorded on a Bruker Vector-22 spectrometer. Samples were prepared as thin films, obtained from chloroform solutions dried on the surface of the KBr tablet. The melting points were measured using the Stuart SMP10.

### 2.2. Reagents

All reagents were purchased from either Acros or Sigma-Aldrich and used without further purification. Solvents were purified according to standard methods [18]. 1-*Iso*propylimidazole [19], 1-(2,6-diisopropylphenyl)imidazole [20], 1-mesitylimidazole [20], 5,11,17,23-tetra-*tert*-butyl-25,26,27,28-tetrahydroxycalix[4]arene [21], 25,26,27,28-tetrahydroxycalix[4]arene [22], 25,27-dihydroxy-26,28-dibutoxycalix[4]arene [23] were synthesized according to published methods.

*11,23-Bis(chloromethyl)-25,27-dihydroxy-26,28-dibutoxycalix[4]arene (**4**)* 5,11,17,23-tetra-H-25,27-dihydroxy-26,28-dibutoxycalix[4]arene (0.67 g (1.22 mmol)) and 0.55 g (18.36 mmol) of paraform were suspended in 10 mL of glacial acetic acid and then bubbled with HCl gas for 2 h. Reaction was stopped by adding 30 mL of water. The crude product was filtered off, washed by 10% acetic acid (3 × 20 mL), water (3 × 20 mL) and dried in vacuo. The final product was obtained as a white solid (0.75 g, 97%). Melting point: 188 °C (decomp). ^1^H NMR (400 MHz, CDCl_3_, 25 °C) δ_H_ ppm: 1.09 (6H, t, CH_3_, *J* = 7.3 Hz,), 1.72–1.83 (4H, m, -CH_2_-), 1.98–2.08 (4H, m, -CH_2_-), 3.38 (4H, d, -CH_2_-, *J* = 12.9 Hz,), 4.00 (4H, t, -CH_2_O-, *J* = 6.35 Hz), 4.29 (4H, d, -CH_2_-, *J* = 12.9 Hz,), 4.51 (4H, s, CH_2_Cl), 6.78 (2H, t, HAr, *J* = 7.6 Hz), 6.93 (4H, d, HAr, *J* = 7.6 Hz), 7.08 (4H, s, HAr), 8.48 (2H, s, OH). ^13^C{^1^H} NMR: (100.6 MHz, CDCl_3_, 25 °C) δ_c_ ppm: 14.22, 19.52, 31.52, 32.36, 47.08, 125.55, 127.87, 128.46, 129.12, 129.18, 133.25. IR (KBr) ν_max_ cm^−1^: 2931 (-CH_2_-), 2959 (-CH_3_), 3318 (OH). MALDI-TOF (*m/z*): 561 [M-2HCl+H]^+^. Elemental analysis calculated (calcd) for C_38_H_42_Cl_2_O_4_: C, 72.03; H, 6.68. found: C, 72.09; H, 6.72.

*General procedure for synthesis of compounds **5**–**9**.*

*N*-substituted imidazole (benzimidazole) (0.8 mmol) and calixarene 4 (0.16 mmol) were dissolved in 3 mL of dry acetonitrile. Resulting mixture was refluxed during 6 h, and then solvent was evaporated in vacuo to give light-yellow oil. Diethyl ether was added to the resulting oil, and the precipitate formed was filtered and dried in a desiccator.

*11,23-Bis[(3-methyl-1H-imidazolium-1-yl)methyl]-25,27-dihydroxy-26,28-dibutoxycalix[4]arene dichloride (**5**)* White solid, 0.10 g, 85%. Melting point: 185 °C (decomp). ^1^H NMR (400 MHz, DMSO-d_6_, 25 °C) δ_H_ ppm: 1.06 (6H, t, CH_3_, *J* = 7.1 Hz), 1.83–1.71 (4H, m, -CH_2_-), 1.88–2.03 (4H, m, -CH_2_-), 3.44 (4H, d, Ar-CH_2_-Ar, *J* = 12.8 Hz), 3.85 (6H, s, CH_3_Im), 3.97 (4H, t, -CH_2_O-, *J* = 6.4 Hz), 4.16 (4H, d, Ar-CH_2_-Ar, *J* = 12.8 Hz), 5.19 (4H, s, Ar-CH_2_N), 6.80 (2H, t, HAr, *J* = 7.3 Hz), 7.05 (4H, d, HAr, *J* = 7.4 Hz), 7.28 (4H, c, HAr), 7.69 (2H, brs, Im), 7.75 (2H, brs, Im), 8.70 (2H, s, OH), 9.18 (2H, s, N-CH-N). ^13^C{^1^H} NMR: (100.6 MHz, CDCl_3_, 25 °C) δ_c_ ppm: 14.21, 19.47, 31.25, 32.31, 34.49, 52.51, 122.39, 122.96, 124.78, 125.48, 128.93, 129.27, 129.52, 133.47, 137.19, 152.24, 154.21. IR (KBr) ν_max_ cm^−1^: 1434 (-CH_3_), 2932 (-CH_2_-), 2959 (-CH_3_), 3376 (OH), 1560 (C = N). MALDI-TOF (*m/z*): 644 [M-C_4_H_6_N_2_-2HCl+H]^+^. Elemental analysis calcd for C_46_H_54_Cl_2_N_4_O_4_: C, 69.25; H, 6.82; N, 7.02, found: 69.32; H, 6.87; N, 6.98.

*11,23-Bis[(3-isopropyl-1H-imidazolium-1-yl)methyl]-25,27-dihydroxy-26,28-dibutoxycalix[4]arene dichloride (**6**)* White solid, 0.10 g, 82%. Melting point: 174 °C (decomp). ^1^H NMR (400 MHz, DMSO-d_6_, 25 °C) δ_H_ ppm: 1.06 (6H, t, CH_3_, *J* = 7.1 Hz), 1.47 (12H, d, CH-CH_3_, *J* = 6.5 Hz), 1.71–1.84 (4H, m, -CH_2_-), 1.90–2.02 (4H, m, -CH_2_-), 3.44 (4H, d, -CH_2_-, *J* = 12.7 Hz), 3.97 (4H, t, CH_2_O, *J* = 6.0 Hz ), 4.16 (4H, d, Ar-CH_2_-Ar, *J* = 12.7 Hz), 4.69–4.59 (2H, sept, CH_3_-CH-CH_3,_ *J* = 6.4 Hz), 5.18 (4H, s, Ar-CH_2_N), 6.76 (2H, t, HAr, *J* = 7.5 Hz), 7.04 (4H, d, HAr, *J* = 7.5 Hz), 7.29 (4H, s, HAr), 7.78 (2H, brs, CH-Im), 7.89 (2H, brs, CH-Im), 8.70 (2H, s, OH), 9.37 (2H, s, N-CH-N). ^13^C{^1^H} NMR: (100.6 MHz, CDCl_3_, 25 °C) δ_c_ ppm: 14.18, 19.44, 22.60, 31.30, 32.26, 53.05, 119.70, 121.88, 123.78, 125.52, 129.10, 129.28, 129.52, 129.79 133.27, 136.01, 152.07, 154.48. IR (KBr) ν_max_ cm^−1^: 2934 (-CH_2_-), 2960 (-CH_3_), 3318 (OH), 1554 (C = N). MALDI-TOF (*m/z*): 672 [M-C_6_H_10_N_2_-2HCl+H]^+^. Elemental analysis calcd for C_50_H_62_Cl_2_N_4_O_4_: C, 70.32; H, 7.32; N, 6.56, found: C, 70.37; H, 7.38; N, 6.51.

*11,23-Bis[(3-(2,6-diisopropylphenyl)-1H-imidazolium-1-yl)methyl]-25,27-dihydroxy-26,28-dibutoxycalix[4]arene dichloride (**7**)* White solid, 0.14 g, 82%. Melting point: 175 °C (decomp). ^1^H NMR (400 MHz, DMSO-d_6_, 25 °C) δ_H_ ppm: 1.07 (6H, t, CH_3_, *J* = 7.4 Hz), 1.13 (12H, d, CH_3_, *J* = 6.4 Hz), 1.17 (12H, d, CH_3_, *J* = 6.5 Hz), 1.72–1.83 (4H, m, -CH_2_-), 1.91–2.02 (4H, m, -CH_2_-), 2.18–2.30 (4H, m, CH-Im), 3.43 (4H, d, Ar-CH_2_-Ar, *J* = 12.6 Hz), 3.99 (4H, s, CH_2_O), 4.20 (4H, d, Ar-CH_2_-Ar, *J* = 12.6 Гц), 5.36 (4H, s, Ar-CH_2_N), 6.77 (2H, t, HAr, *J* = 7.3 Hz), 7.02 (4H, d, HAr, *J* = 7.4 Hz), 7.29 (4H, s, HAr), 7.47 (4H, д, HAr-Im, *J* = 7.7 Гц), 7.64 (2H, т, HAr-Im), 8.06 (2H, s, CH-Im), 8.09 (2H, s, CH-Im), 8.71 (2H, s, OH), 9.84 (2H, s, N-CH-N). ^13^C{^1^H} NMR: (100.6 MHz, CDCl_3_, 25 °C) δ_c_ ppm: 14.21, 19.47, 24.21, 24.38, 28.78, 31.34, 32.31, 53.63, 122.61, 123.38, 123.62, 124.03, 124.75, 125.53, 129.23, 129.80, 131.96, 133.08, 138.33, 145.34, 151.99, 154.59. IR (KBr) ν_max_ cm^−1^: 2934 (-CH_2_-), 2960 (-CH_3_), 3318 (OH), 1544 (C = N), 1484 (C = C). MALDI-TOF (*m/z*): 790 [M-C_15_H_20_N_2_-2HCl+H]^+^. Elemental analysis calcd for C_68_H_82_Cl_2_N_4_O_4_: C, 74.91; H, 7.58; N, 5.14, found: C, 74.96; H, 7.56; N, 5.11.

*11,23-Bis[(3-(mesityl)-1H-imidazolium-1-yl)methyl]-25,27-dihydroxy-26,28-dibutoxycalix[4]arene dichloride (**8**)* White solid, 0.15 g, 85%. Melting point: 172 °C (decomp). ^1^H NMR (400 MHz, DMSO-d_6_, 25 °C) δ_H_ ppm: 1.07 (6H, t, CH_3_, *J* = 7.2 Hz), 1.72–1.83 (4H, m, CH_2_), 1.90–2.07 (16H, m, CH_3_Im, CH_2_), 2.33 (6H, s, CH_3_Im), 3.45 (4H, d, Ar-CH_2_-Ar, *J* = 12.8 Hz), 3.97 (4H, t, CH_2_O, *J* = 5.8 Hz), 4.18 (4H, d, Ar-CH_2_-Ar, *J* = 12.7 Hz), 5.31 (4H, s, Ar-CH_2_-N), 6.75 (2H, t, HAr, *J* = 7.5 Hz), 7.03 (4H, d, HAr, *J* = 7.5 Hz), 7.16 (4H, s, HAr), 7.33 (4H, s, HAr-Im), 7.88 (2H, s, CHIm), 8.00 (2H, s, CHIm), 8.72 (2H, s, OH), 9.62 (2H, s, N-CH-N). ^13^C{^1^H} NMR: (100.6 MHz, CDCl_3_,25 °C) δ_c_ ppm: 14.22, 17.70, 19.49, 21.19, 31.37, 32.33, 53.61, 122.25, 122.97, 123.70, 125.60, 129.21, 129.28, 129.79, 129.95, 130.89, 133.06, 134.24, 138.31, 141.31, 151.98, 154.60. IR (KBr) ν_max_ cm^−1^: 1553 (C = N), 2933 (CH_2_), 2959 (CH_3_), 3318 (OH). MALDI-TOF (*m/z*): 748 [M-C_12_H_14_N_2_-2HCl+H]^+^, 562 [M-2C_12_H_14_N_2_-2HCl+H]^+^. Elemental analysis calcd for C_62_H_70_Cl_2_N_4_O_4_: C, 74.01; H, 7.01; N, 5.57, found: C, 74.07; H, 7.05; N, 5.59.

*11,23-Bis[(3-methyl-1H-benzimidazolium-1-yl)methyl]-25,27-dihydroxy-26,28-dibutoxycalix[4]arene dichloride (**9**)* White solid, 0.08 g, 56%. Melting point: 167 °C (decomp). ^1^H NMR (400 MHz, DMSO-d_6_, 25 °C) δ_H_ ppm: 1.04 (6H, t, CH_3_, *J* = 6.7 Hz), 1.70–1.80 (4H, m, CH_2_), 1.88–1.98 (4H, m, CH_2_), 3.43 (4H, d, Ar-CH_2_-Ar, *J* = 12.8 Hz), 3.95 (4H, brt, CH_2_O), 4.06–4.18 (10H, m, Ar-CH_2_-Ar, CH_3_Im), 5.53 (4H, s, Ar-CH_2_-N), 6.76 (2H, t, HAr, *J* = 7.2 Hz), 7.01 (4H, d, HAr, *J* = 7.4 Hz), 7.39 (4H, s, HAr), 7.68 (4H. brs, HAr-Im), 8.02 (4H, m, HAr-Im), 8.67 (2H, s, OH), 9.88 (2H, s, N-CH-N). ^13^C{^1^H} NMR: (100.6 MHz, CDCl_3_, 25 °C) δ_c_ ppm: 14.18, 19.44, 31.28, 32.28, 32.57, 51.13, 112.62, 114.01, 123.66, 125.51, 127.15, 129.06, 129.53, 130.92, 131.95, 133.30, 143.02, 152.02, 154.31. IR (KBr) ν_max_ cm^−1^: 1485 (C = C), 1567 (C = N), 2933 (CH_2_), 2959 (CH_3_), 3334 (OH). MALDI-TOF (*m/z*): 861 [M-Cl]^+^, 694 [M-C_8_H_8_N_2_-2HCl+H]^+^. Elemental analysis calcd for C_54_H_58_Cl_2_N_4_O_4_: C, 72.23; H, 6.51; N, 6.24, found: C, 72.29; H, 6.58; N, 6.27.

### 2.3. Dynamic Light Scattering and Zeta-Potential

Dynamic light scattering (DLS) experiments and zeta-potential measurements were carried out on the Zetasizer Nano ZS instrument (Malvern Instruments, Worcestershire, UK) with 4 mW 633 nm He–Ne laser light source and the light scattering angle of 173°. The data were treated with DTS software (Dispersion Technology Software 5.00). The solutions were filtered through a 0.8 μM filter before the measurements to remove dust. The experiments were carried out in the disposable plastic cells DTS 0012 (size) or in the disposable folded capillary cells DTS 1070 (zeta potential) (Sigma–Aldrich, St. Louis, MO, USA) at 298 K with at least three experiments for each system. Statistical data treatment was done using t-Student coefficient and the particle size determination error was <2%. The prepared samples were ultra-sonicated within 30 min at 25 °C before measurements.

### 2.4. Critical Aggregation Concentration Determination

Critical aggregation concentration (CAC) values were measured using pyrene fluorescent probe and calculated from the dependence of the intensity ratio of the first (373 nm) and third (384 nm) bands in the emission spectrum of pyrene vs. calixarene concentration. Fluorescence experiments with pyrene were performed in 10.0 mm quartz cuvettes and recorded on a Fluorolog FL-221 spectrofluorimeter (HORIBA Jobin Yvon, Edison Township, NJ, USA) in the range of 350 to 430 nm and excitation wavelength 335 nm with 2.5 nm slit. All studies were conducted in buffered aqueous solution (TRIS buffer, pH 7.4) at 298 K.

### 2.5. Vesicles Preparation

DPPC lipid films were formed from chloroform solutions, dried at 85 °C, and left under reduced pressure for a minimum of 2 h to remove all traces of organic solvent. Turbid MLV-suspensions were prepared by adding 1.15 mL of water Milli-Q water to the films and heating for 1 h at 65 °C. SUV-dispersions were obtained by extrusion through 100 nm-pore size polycarbonate membranes with a Mini-Extruder from Avanti. Concentration of DPPC stock dispersion was typically 1 mM. For binary system with calixarene 43, 65 or 98 µM of **5** were added to the chloroform solutions during preparation the lipid films.

### 2.6. Turbidity Measurements

The dependence of optical density at wavelengths of 400 nm on temperature was recorded using a Shimadzu UV-2600 spectrophotometer equipped with a Shimadzu TCC-100 thermostat. The temperature was varied in the range from 25 to 55 °C, the heating rate was 0.1 °C/min. The obtained plots were mathematically treated using Van’t-Hoff’s two-state model. [24] In accordance with this model the main temperature of phase transition of the lipid bilayer corresponds to the inflection point of the respective turbidity plot.

### 2.7. Gas Chromatography Mass Spectrometry

Gas chromatography mass spectrometry was performed on a GCMS-QP2010 Ultra gas chromatography mass spectrometer (Shimadzu, Kyoto, Japan) equipped with an HP-5MS column (the internal diameter was 0.25 μm and the length was 30 m). The parameters were as follows: Helium A was the carrier gas, the temperature of an injector was 250 °C, the flow rate through the column was 2 mL/min, the thermostat temperature program was a gradient temperature increase from 70 to 250 °C with a step of 10 °C/min. The range of scanned masses was *m*/*z* 35 ÷ 400. The internal standard method using dodecane was used for the quantitative analysis of 4-nitro-1,1’-biphenyl.

### 2.8. Suzuki–Miyaura Coupling

Reactions were performed in 2 mL Pyrex vials in IKA heating block with vigorous stirring. Aqueous dispersion (1 mL) containing 50 µM Pd(OAc)_2_, **5**–**9** (0.098, 0.033, 0.060, 0.080 or 0.18 mM for **5**–**9**, respectively) or DPPC-**5** vesicles was filled with 10 mM 1-bromo-4-nitrobenzene, 11 mM phenylboronic acid, 1 mM of dodecane and 30 mM K_2_CO_3_ (30 mM). The mixture was degassed with Ar for 10 min by piercing the septum of the vial with two needles for supplying and discharging gas. The mixture was stirred at 70 °C. Two aliquots (0.1 mL) for GCMS analysis were taken from the reaction mixture after 1 and 4 h and then extracted with chloroform (3 × 0.5 mL).

## 3. Results and Discussion

### 3.1. Synthesis of Imidazolium/Benzimidazolium Calix[4]arene Derivatives

As it was previously shown by Schatz group, tetra-O-alkyl calixarene derivatives bearing at the distal positions of upper rim two [(3-*N*-substituted imidazolium-1-yl)methyl] fragments formed stable *N*-heterocyclic carbenes and corresponding cis/trans Pd(II) complexes which effectively catalyzed Suzuki–Miyaura coupling [25,26]. This structural motive of NHC precursors with two imidazolium fragments on the upper rim seems to be very attractive for designing self-organizing systems for different catalytic transformations in water. The preparation of calixarene imidazolium salts was based on the tetra-O-propyl substituted dichloromethyl calix[4]arene as precursor. However, its synthesis is rather complicated and included at least seven stages starting from parent *p-tert-*butyl calix[4]arene (Scheme S1 in Supplementary Materials) [23,27].

**Scheme 1:**
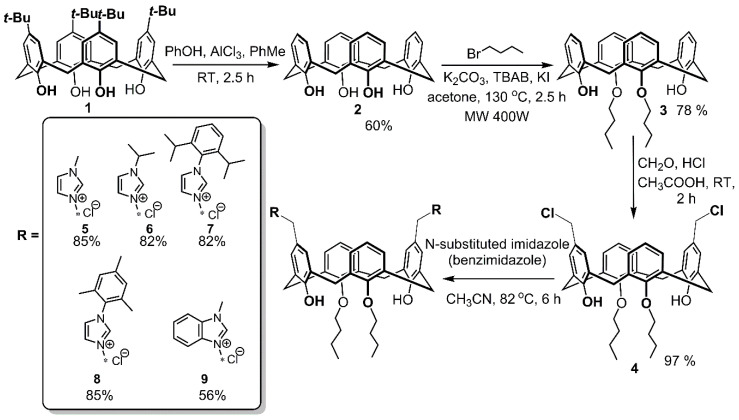
Synthetic pathway for imidazolium/benzimidazolium salts **5**–**9**.

To design amphiphilic calixarene imidazolium salts we suggest a more synthetically accessible precursor containing two (instead of four) O-alkyl groups on the macrocycle lower rim (**3**, Scheme 1). In this case the number of stages for the target product is halved (4 vs. 8). The application of distal di-O-alkyl calixarene derivative **3** (Scheme 1) allows to introduce selectively two chloromethyl substituents on the upper rim in one step due to significantly different reactivity of *p*-positions of aromatic rings containing OH and OAlk groups [28]. Taking into account that tetra-O-alkyl substituted bis-imidazolium calixarene has a very low solubility in water [29], a decrease of the number of alkyl groups on the calixarene lower rim (2 vs. 4) gives another very important advantage for the applications in aqueous solutions: Better solubility in water.

Parent *p-tert-*butyl calix[4]arene **1** was de-*tert*-butylated using the earlier described procedure [22]. Obtained *p*-H-calix[4]arene **2** was treated by butyl bromide under microwave irradiation and O,O- dialkyl derivative **3** was formed. Then it was involved in the Blanc reaction to give dichloromethyl macrocycle **4** with nearly quantitative yield. The structure of **4** was established by NMR ^1^H and ^13^C spectroscopy as well as IR spectroscopy and MALDI-TOF mass spectrometry (see Supplementary Materials). The composition was determined by the element analysis. A set of signals which are characteristic for distal chloromethylated calixarene was found in the NMR ^1^H spectra of **4**: A singlet of chloromethylene protons at 4.51 ppm, signals of aromatic calixarene protons as singlet, doublet and triplet at 7.08, 6.93 and 6.78 ppm, respectively.

**Figure 1:**
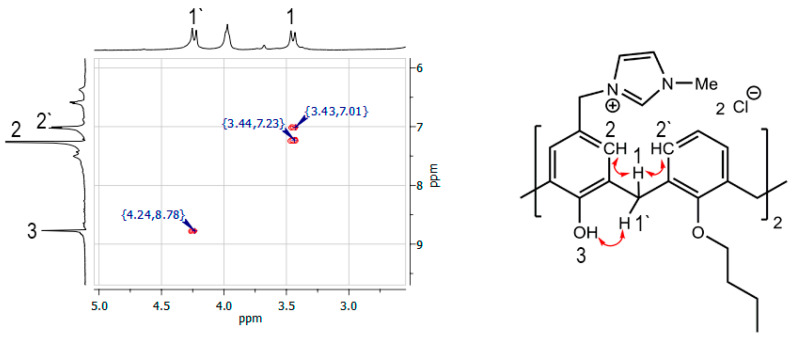
Fragment of the ^1^H-^1^H NOESY NMR spectra of **5** (CDCl_3_).

Chloromethylated calixarene **4** was functionalized by imidazolium/benzimidazolium moieties. Macrocycle **4** was heated with five-fold excess of appropriate *N*-substituted imidazole/benzimidazole in acetonitrile for 6 h. Corresponding imidazolium salts **5**–**8** were obtained with high yields (82–85%). The lower yield of benzimidazolium salt **9** (56%) can be attributed to its slightly better solubility in diethyl ether, which was used to precipitate compound compared to that for imidazolium salts **5**–**8**. The structures of **5**–**9** were well-established with the same set of methods used for **4**. In all synthesized salts the singlet of the methylene spacer between macrocycle and heterocycle cores is downshifted to Δδ ~0.7–1 ppm in comparison to **4** due to the effect of a positive charge of imidazolium/benzimidazolium fragments and the location of the methylene spacer in the deshielding region of the imidazolium/benzimidazolium molecular ring current. Moreover, *N*-substituted imidazolium/benzimidazolium fragments appeared as a standard set of signals: Two broad singlets of heterocycle core at 7.7–8.0 and 7.8–8.1 ppm and one singlet of NCHN protons at 9.2–9.9 ppm. Interestingly, molecular ion peaks with expulsion of one imidazolium/benzimidazolium fragment and two molecules of HCl were observed in MALDI-TOF spectra of salts **5**–**9** due to easy formation of *p*-quinone methide structures [30]. The stereoisomeric form of **5**–**9** can be easily identified using the Gutsche’s “1H NMR ∆δ” rule [31]: Chemical shift separation of ArCH_2_Ar protons ∆δ ≈ 0.7–1.0 is indicative of a *syn* orientation of the two pertinent aromatic rings, typical of the *cone* stereoisomeric form. Indeed, in all NMR spectra of **5**–**9** ∆δ of signals of ArCH_2_Ar protons are 0.72–0.76 ppm indicating thus the *cone* stereoisomeric form. Additionally, the stereoisomeric form of macrocycle **5** was established using two-dimensional ^1^H-^1^H NOESY NMR spectroscopy (Figure 1).

The presence of a cross peak between equatorial ArCH_2_Ar protons with protons of hydroxyl group (δ = 4.24 and 8.78 ppm) as well as cross peaks between axial ArCH_2_Ar protons and aryl protons (δ = 3.44 and 7.23, 7.01 ppm) clearly indicates the *cone* stereoisomeric form of **5**.

### 3.2. Aggregation Behavior of 5–9 in Aqueous Solutions

The aggregates formation in the aqueous solutions is typical behavior for calix[4]arenes containing positively charged headgroups and alkyl moieties on the upper and lower rim, respectively [32,33]. To estimate amphiphilic properties of calixarenes **5**–**9** their critical aggregation concentration (CAC) values were measured using a pyrene fluorescent probe.

**Figure 2:**
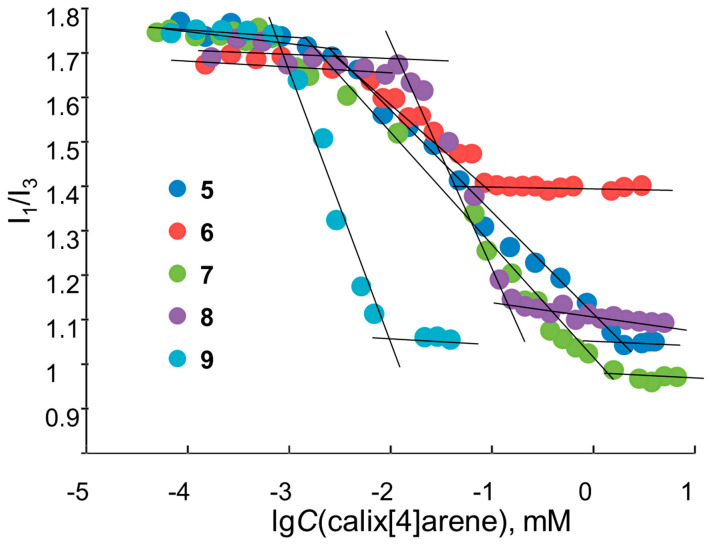
Dependence of I_1_/I_3_ pyrene ratio vs. lg(C) of **5**–**9**, C (pyrene) = 1 µM in ultrapure water, 25 °C.

The intensity ratio changes of the first and third peaks of pyrene (so called polarity index [34]) (Figure 2) indicate a decrease in the pyrene polarity environment caused by its solubilization in the hydrophobic core of the calixarene aggregates. Calculated CAC values are given in Table 1.

It was found that, in the case of imidazolium-containing calixarenes (**5**–**8**), CAC values were not sensitive to the *N*-substituents of the heterocycle. Neither nature (alkyl or aryl) or size (steric hindrance) of *N*-substituents had no effect on the CAC values (50 ± 10 µM). This can be explained by the fact that *N*-substituents do not participate in the aggregate hydrophobic core formation because they are constrained to orientate into the aqueous phase for steric reasons. On other hand the aromatic ring condensed at 4,5-positions of imidazole (benzimidazolium calixarene **9**) increased the CAC values by more than double. Such a difference in CAC values between imidazole and benzimidazole derivatives may be associated with the delocalization of the positive charge on the entire aromatic benzimidazole system, while in the case of imidazolium compounds it concentrates in the imidazole cycle [35]. Thus, in the case of benzimidazole, the charged groups occupy a larger volume, which leads to an increase of electrostatic repulsion between molecules during the aggregation process, and, as a result, increases CAC.

Additionally, the study of aggregation properties was carried out using dynamic (DLS) and electrophoretic (ELS) light scattering methods (Table 1) at a concentration above corresponding CAC of macrocycles (1.5× CAC µM). All macrocycles formed submicron particles in aqueous solution with a hydrodynamic diameter in the range of 340–420 nm. Electrokinetic potential of the aggregates corresponds to the positive charge of the imidazolium/benzimidazolium headgroups and is about +44–+57 mV that refers them to the stable colloids. However, a rather high polydispersity index was indicated on a wide size distribution of aggregates. This is clearly seen from the DLS curves (Supplementary Materials).

### 3.3. Complexes of 5–9 with Pd(II) Obtained In Situ in Model Suzuki–Miyaura Coupling

**Figure 3:**
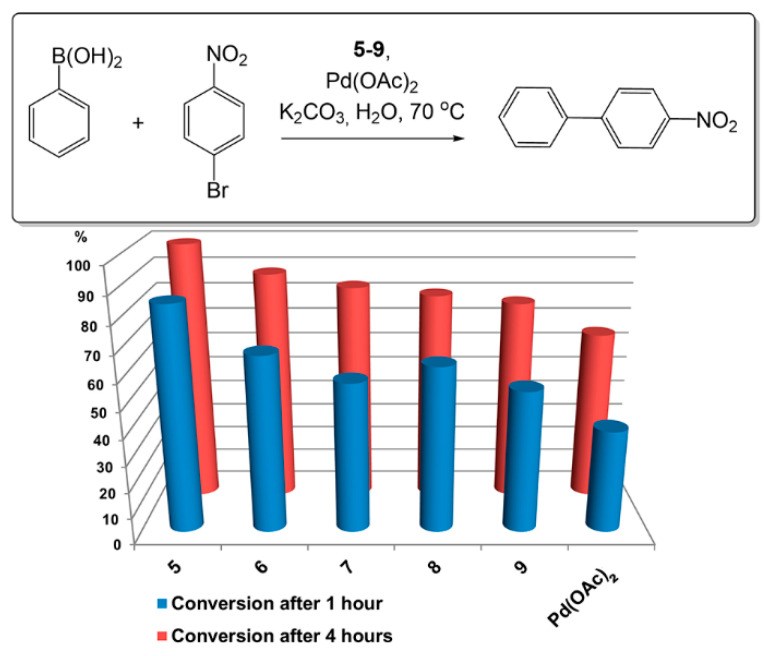
Conversion of 1-bromo-4-nitrobenzene after 1 or 4 h of reaction upon reaction with phenylboronic acid. *C*(1-bromo-4-nitrobenzene) = 10 mM, *C*(phenylboronic acid) = 11 mM, *C*(K_2_CO_3_) = 30 mM, *C*(Pd(OAc)_2_) = 50 µM, C(**5**–**9**) = 1.5× CAC µM, H_2_O, 70 °C.

NHC-Pd complexes are well-known catalysts [36] in Suzuki–Miyaura coupling [1]. Obviously, many catalytic systems have been applied for this reaction including in situ systems, generated from appropriate imidazolium salts and Pd(OAc)_2_ [37]. The attempts to isolate and characterize corresponding NHC complexes were unsuccessful because they decomposed easily during column chromatography. For this reason, catalytic activity of in situ prepared complexes of amphiphilic calixarenes **5**–**9** and Pd(OAc)_2_ was investigated in Suzuki–Miyaura coupling of 1-bromo-4-nitrobenzene with phenylboronic acid in water (Figure 3). Reactions were performed in the presence of 0.5 mol% of Pd(OAc)_2_ and K_2_CO_3_ as a base at 70 °C. The concentration of calixarenes **5**–**9** was one and a half times greater than their CAC values to form aggregates in the solution for effective substrate solubilization. Conversion of haloarenes and 4-nitro-1,1’-biphenyl formation selectivity were evaluated using GCMS with dodecane as the internal standard.

It is important that selectivity of 4-nitro-1,1’-biphenyl formation was very high (96–99%) for all investigated catalytic systems. Calixarene containing catalytic systems showed a higher activity than pure Pd(OAc)_2_ (Figure 3). The influence of alkyl/aryl substituent in N-position on catalytic activity in the series of calixarenes **5**–**9** was evaluated. Compound **5** with methyl substituent was found to be the most active—conversion of 1-bromo-4-nitrobenzene was achieved at 80% and 98% after 1 and 4 h, respectively. Compounds with more bulky isopropyl (**6**), mesityl (**7**) and 2,6-diisopropylphenyl (**8**) substituents in the N-position of the imidazolium unit demonstrated lower conversion of 1-bromo-4-nitrobenzene: 50–60% and 75–85% after 1 and 4 h, respectively. The benefits of using calixarenes **5**–**9** with Pd(OAc)_2_ are more apparent when comparing turnover number (TON) and turnover frequency (TOF) values (Table 2). According to this data, calixarenes give at least a 2.5-fold increase in TON and TOF, and macrocycle **5** shows better results and gives a four-fold increase.

These results are not consistent with the usual effect of the bulky substituents on the catalytic activity of pre-prepared NHC complexes: Usually bulky groups promote the last reductive elimination step of Suzuki–Miyaura coupling as well as the formation of active Pd(0) species through favorable repulsion of halides from the coordination sphere of Pd(II) [38,39]. However, it can be assumed that catalytically active species in the case of less bulky NHC forms faster. In confirmation of this, recently we showed that the reaction time of bis-imidazolium thiacalix[4]arenes with Pd(OAc)_2_ to give NHC–Pd(II) complexes increases from 28 to 60 h upon going from methyl to the more bulky mesityl and 2,6-diisopropylphenyl substituents in the N-position of the imidazole [40]. Thus, lower rates of the NHC–Pd(II) complex in situ formation could be a reason for their lower activity in Suzuki–Miyaura coupling. The lower activity of benzimidazole derivative **9** can be attributed to a decrease in the relative stability of the in situ complex, which correlates with the basicity of NHC ligands and is decreased going from *N*-methylimidazole to *N*-methylbenzimidazole [41].

### 3.4. Embedding of 5 into DPPC Vesicles and Their Catalytic Activity in Suzuki–Miyaura Coupling

An alternative approach to the application of amphiphilic compounds containing receptor groups for the catalytic systems design is their embedding into vesicles and liposomes, which have a well-defined size/shape and can act as microreactors. This is a well-known and very promising way to increase the reaction rates and to solubilize hydrophobic reagents [42,43]. So, metallocomplex and micellar catalysis can be combined in one catalytic system.

Recently, phospholipid vesicles were successfully used as both effective stabilizers for Pd(0) nanoparticles and microreactors for Suzuki–Miyaura coupling in aqueous media [44]. To the best of our knowledge, vesicles doped by amphiphilic NHC metal complexes and their application for the catalysis are not presented in literature.

In our investigation DPPC (1,2-dipalmitoyl-sn-glycero-3-phosphocholine) liposomes were chosen as a typical membrane mimicking system, which is characterized by close to zero zeta potential and a hydrodynamic diameter of ca. 100 nm. As the amphiphilic guest molecule, macrocycle **5** was used because it demonstrated the best catalytic activity among synthesized compounds. The vesicles modified by amphiphilic calixarene were obtained by the film-hydration method [40] from a mixture of DPPC and calixarene **5** chloroform solutions (from 0.04 up to 0.10 calixarene/DPPC molar ratio). Size, polydispersity index and electrokinetic potential of mixed phospholipid vesicles before and after extrusion are presented in Table 3.

It can be seen that the size of the DPPC multilamellar vesicles (MLVs) before extrusion is 600 ± 63 nm with high PDI values. The extrusion process leads to the transformation of MLVs into unilamellar ones (SUVs) with a narrow size distribution (106 ± 2 nm, PDI 0.078). For the first time it was found that addition of amphiphilic calixarene leads to the same effect. DPPC multilamellar vesicles were easily destroyed and transformed into unilamellar ones in the presence of macrocycle **5**. Moreover, amphiphile concentration plays a major role in this process. At the small calixarene/DPPC molar ratio (0.04) the aggregates size almost decreased five times. At the higher ratio, the size and PDI values of obtained aggregates before and after extrusion became practically equal. Evidently, the embedding of a positively charged macrocycle into the multilamellar vesicles leads to an “explosion” of huge particles due to Coulomb repulsion between charged monolayers. Of course, the size and shape of the amphiphile may play a key role as well. The commonality and reasons of such behavior are now under investigation. Nevertheless, it can be concluded from DLS data that calixarene **5** was embedded into DPPC vesicles, thus forming mixed nanosized vesicles, since both calixarene and DPPC separately form larger aggregates.

The gel-to-liquid crystalline phase transition, named the main phase transition, is an informative parameter that is sensitive to guest molecules. In particular, a change of the main phase transition temperature, the so-called melting point T_m_, indicates that a perturbation of the lipid organization occurs, and so indicates the incorporation of guest molecules into the lipid bilayer. The T_m_ value can be monitored by the turbidimetry technique, a well-known method of detection of this parameter with high reproducibility [24]. Turbidity curves and T_m_ values for DPPC+**5** systems are presented on Figure S9 (Supplementary Materials).

**Figure 4:**
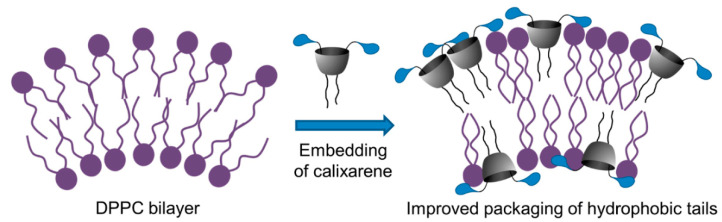
Schematic representation of improved packaging of hydrophobic tales in mixed DPPC–calixarene **5** vesicles.

For a single DPPC bilayer Tm is ca. 41 °C [45], while the introduction of surfactants may change this value. Usually a decrease of T_m_ indicates that the macrocyclic amphiphile disorders the lipid bilayer due to the interaction with lipids and promotes the gel-to-liquid phase transition [46]. In our case T_m_ values did not change (~40.9 °C) up to a molar calixarene/DPPC ratio of 0.07, after which the increase of calixarene concentration promotes the ordering of the lipid in membranes (Figure 4), which is in line with an increase in the T_m_ value (43.3 °C). An increase in the temperature of the main phase transition, correlated with the stabilizing effect on the lipid packing, was demonstrated earlier [47] and was explained in terms of an increase in the distribution coefficient value for amphiphile in the gel phase in comparison with the distribution coefficient in the liquid phase.

Preliminarily, to estimate the catalytic effect of amphiphilic calixarene embedding into the vesicles, a blank experiment was carried out. It was found that the catalytic activity of Pd(II) in the presence of DPPC vesicles increases by 20% (Figure 4), which may be assigned to both the stabilization of catalytically active palladium species by DPPC phosphonate groups and/or the micellar catalytic effect due to solubilization and concentration of reagents inside the vesicle.

**Figure 5:**
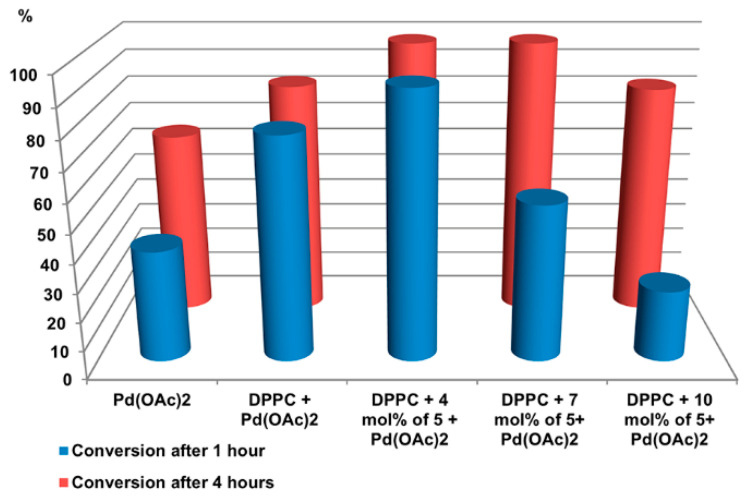
Conversion of 1-bromo-4-nitrobenzene after 1 or 4 h of reaction. *C*(1-bromo-4-nitrobenzene) = 10 mM, *C*(phenylboronic acid) = 11 mM, *C*(K_2_CO_3_) = 30 mM, *C*(DPPC) = 1 мM, C(**5** mixed with DPPC) = 43, 65 and 98 µM, *C*(Pd(OAc)_2_) = 50 µM, H_2_O, 70 °C.

Embedding of calixarene **5** into the vesicles at the small calixarene/DPPC molar ratio (up to 0.07) gave practically quantitative reaction yield after 4 h. Surprisingly, further growth of calixarene concentration in the reaction mixture led to the decrease in the yield. However, according to the turbidity data, an ordering of the lipid in membranes at a larger calixarene/DPPC molar ratio was observed and the lower catalytic activity of such vesicles can be explained by the fact that the better packing of hydrophobic tails in a lipid bilayer prevents free diffusion of reagents inside vesicle. Moreover, the significant effect of the calixarene/DPPC molar ratio on the differences of 1-bromo-4-nitrobenzene conversion for 1 and 4 h indicates diffusion reaction control (Figure 5).

## 4. Conclusions

In this study, new types of amphiphilic calix[4]arene derivatives bearing N-alkyl/aryl imidazolium/benzimidazolium fragments were synthesized according to a two step scheme including regioselective chloromethylation of distal di-O-butyl calix[4]arene and subsequent interaction with *N*-substituted imidazole/benzimidazole. Obtained amphiphilic macrocycles are formed submicron particles with electrokinetic potential +44–+57 mV in aqueous solution. For the first time it was found that amphiphilic calixarene causes the fast transformation of DPPC multilamellar vesicles into unilamellar ones and leads to the ordering of the lipid into membranes at a molar calixarene/DPPC ratio more than 0.07.

In situ complexes of calixarene aggregates with Pd(OAc)_2_ were found to be active in Suzuki–Miyaura coupling of 1-bromo-4-nitrobenzene with phenylboronic acid in water. It was shown that the bulky *N*-substituents of a heterocycle decrease the catalytic activity of the aggregates. These results are not consistent with the usual influence of the bulky substituents on the catalytic activity of pre-prepared NHC complexes and can be assigned to the inhibition effect of Pd(II) complex formation by bulky substituents located on the aggregate surface.

An alternative approach to the catalytic application of amphiphilic receptor molecules based on their embedding into liposome membrane was investigated. Amphiphilic calixarene embedding into DPPC vesicles enhances the catalytic activity in Suzuki–Miyaura coupling.

## Figures and Tables

**Table 1:** Critical aggregation concentration (CAC) values, dynamic light scattering (DLS) and electrophoretic (ELS) data of aggregates formed by macrocycles **5**–**9**.

Calixarene *	CAC, µM	d, nm	PDI	ζ. mV
**5**	65	340 ± 13	0.426 ± 0.081	+57 ± 2
**6**	45	351 ± 51	0.477 ± 0.042	+45 ± 1
**7**	40	420 ± 5	0.473 ± 0.055	+52 ± 1
**8**	53	390 ± 2	0.513 ± 0.030	+44 ± 1
**9**	120	410 ± 18	0.416 ± 0.070	+54 ± 0.5

* Pyrene concentration is 1 µM, C(**5**–**9**) = 1.5× CAC µM.

**Table 2:** (TON) and turnover frequency (TOF) values for coupling of 1-bromo-4-nitrobenzene with phenylboronic acid using different catalytic systems.

Catalyst *	TON	TOF 10^−2^ s^−1^
Pd(OAc)_2_	22	1.8
**5**+Pd(OAc)_2_	89	7.4
**6**+Pd(OAc)_2_	67	5.6
**7**+Pd(OAc)_2_	56	4.6
**8**+Pd(OAc)_2_	62	5.2
**9**+Pd(OAc)_2_	53	4.4

* Values were calculated using conversion after 20 min of reaction, C(1-bromo-4-nitrobenzene) = 10 mM, C(phenylboronic acid) = 11 mM, C(K_2_CO_3_) = 30 mM, C(Pd(OAc)_2_) = 50 µM, C(**5**–**9**) = 1.5× CAC µM, H_2_O, 70 °C.

**Table 3:** DLS and ELS data of aggregates formed by DPPC and calixarene **5**.

System	Calixarene/DPPC Molar Ratio	d, nm		PDI		ζ. mV
		before Extrusion	after Extrusion	before Extrusion	after Extrusion	
DPPC	0	600 ± 63	106 ± 2	0.780 ± 0.140	0.078 ± 0.006	-
DPPC+ **5**	0.04	130 ± 5	64 ± 5	0.703 ± 0.049	0.269 ± 0.074	+22 ± 1
DPPC+ **5**	0.07	60 ± 1	51 ± 2	0.331 ± 0.015	0.239 ± 0.015	+36 ± 3
DPPC+ **5**	0.1	79 ± 1	63 ± 1	0.365 ± 0.011	0.285 ± 0.025	+35 ± 5

## Supplementary Materials

The following are available online at https://www.mdpi.com/2079-4991/10/6/1143/s1, Figures S1–S6: NMR ^1^H, ^13^C and MALDI-TOF spectra of new compounds, Figure S7: DLS size graph of aggregates formed by **5**–**9**, Figure S8: DLS size graph of aggregates formed DPPC or DPPC mixed with **5**, Figure S9: T_m_ plots of DPPC and DPPC-5 vesicles, Scheme S1: Known synthetic pathway for NHC-precursors made by Schatz group.
