# Supplementary material for: New Amphiphilic Imidazolium/Benzimidazolium Calix[4]arene Derivatives: Synthesis, Aggregation Behavior and Decoration of DPPC Vesicles for Suzuki Coupling in Aqueous Media
Source: Nanomaterials (Basel). 2020 Jun 10;10(6):1143. doi: 10.3390/nano10061143 (PMC7353316; doi:10.3390/nano10061143)
Supplement: Supplementary file 1 [file nanomaterials-10-01143-s001.pdf]

Table of contents

1. NMR ^1H, ^{13}C and MALDI-TOF spectra of **4** **2**
2. NMR ^1H, ^{13}C and MALDI-TOF spectra of **5** **3**
3. NMR ^1H, ^{13}C and MALDI-TOF spectra of **6** **4**
4. NMR ^1H, ^{13}C and MALDI-TOF spectra of **7** **5**
5. NMR ^1H, ^{13}C and MALDI-TOF spectra of **8** **6**
6. NMR ^1H, ^{13}C and MALDI-TOF spectra of **9** **7**
7. DLS size graph of aggregates formed by **5-9** **9**
8. DLS size graph of aggregates formed by DPPC or DPPC mixed with **5** **11**
9. Known synthetic pathway for NHC-precursors made by Schatz group. **11**
10. T_m plots of DPPC and DPPC-**5** vesicles **11**

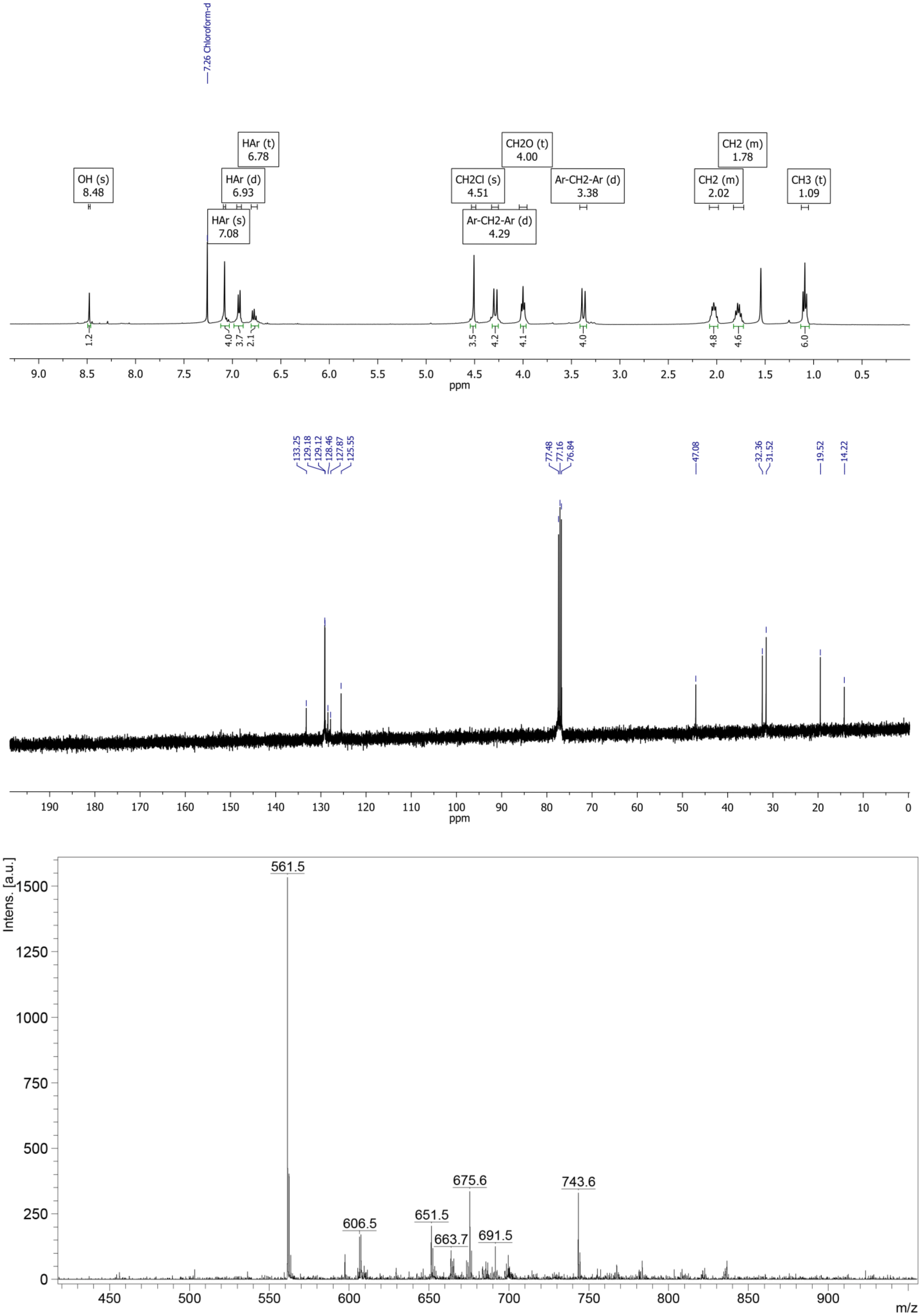

Fig. S1 NMR ^{1}H, ^{13}C and MALDI-TOF spectra of 11,23-Di-chloromethyl-25,27-dihydroxy-26,28-dibutoxycalix[4]arene (**4**)

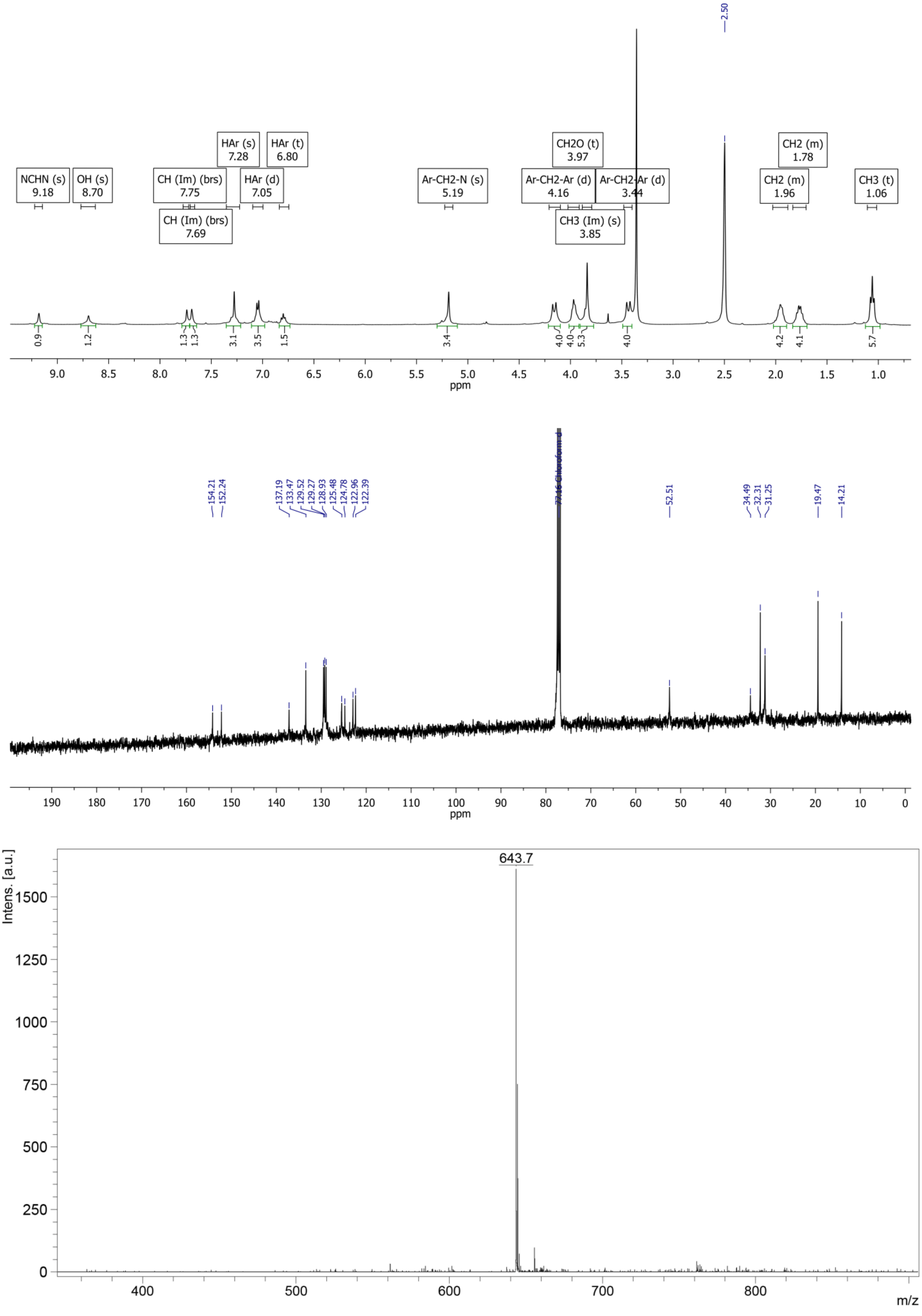

Fig. S2 NMR ^{1}H, ^{13}C and MALDI-TOF spectra of 11,23-bis[(3-methyl-1H-imidazolium-1-yl)methyl]-25,27-dihydroxy-26,28-dibutoxycalix[4]arene dichloride (**5**)

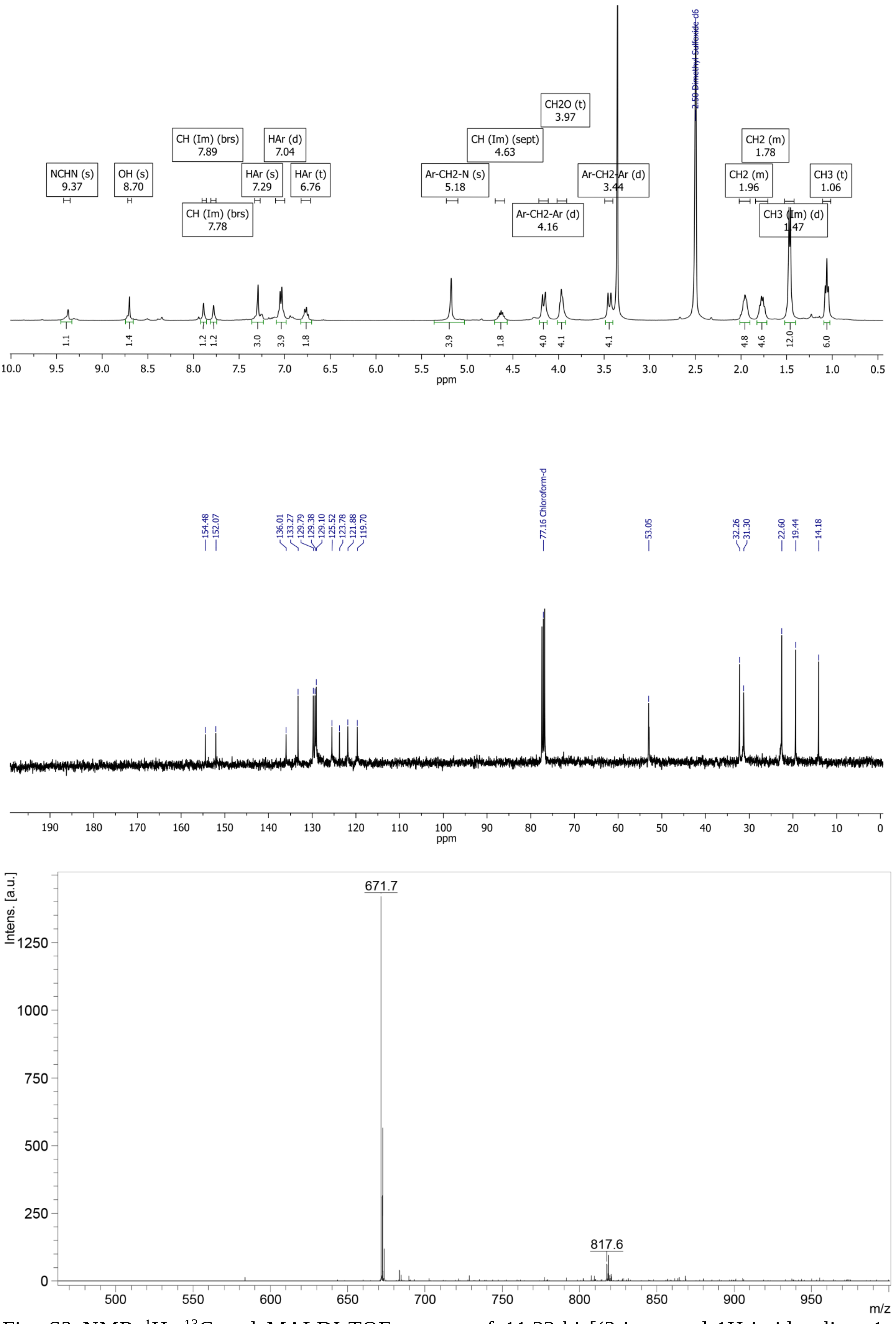

Fig. S3 NMR ^{1}H, ^{13}C and MALDI-TOF spectra of 11,23-bis[(3-isopropyl-1H-imidazolium-1-yl)methyl]-25,27-dihydroxy-26,28-dibutoxycalix[4]arene dichloride (**6**)

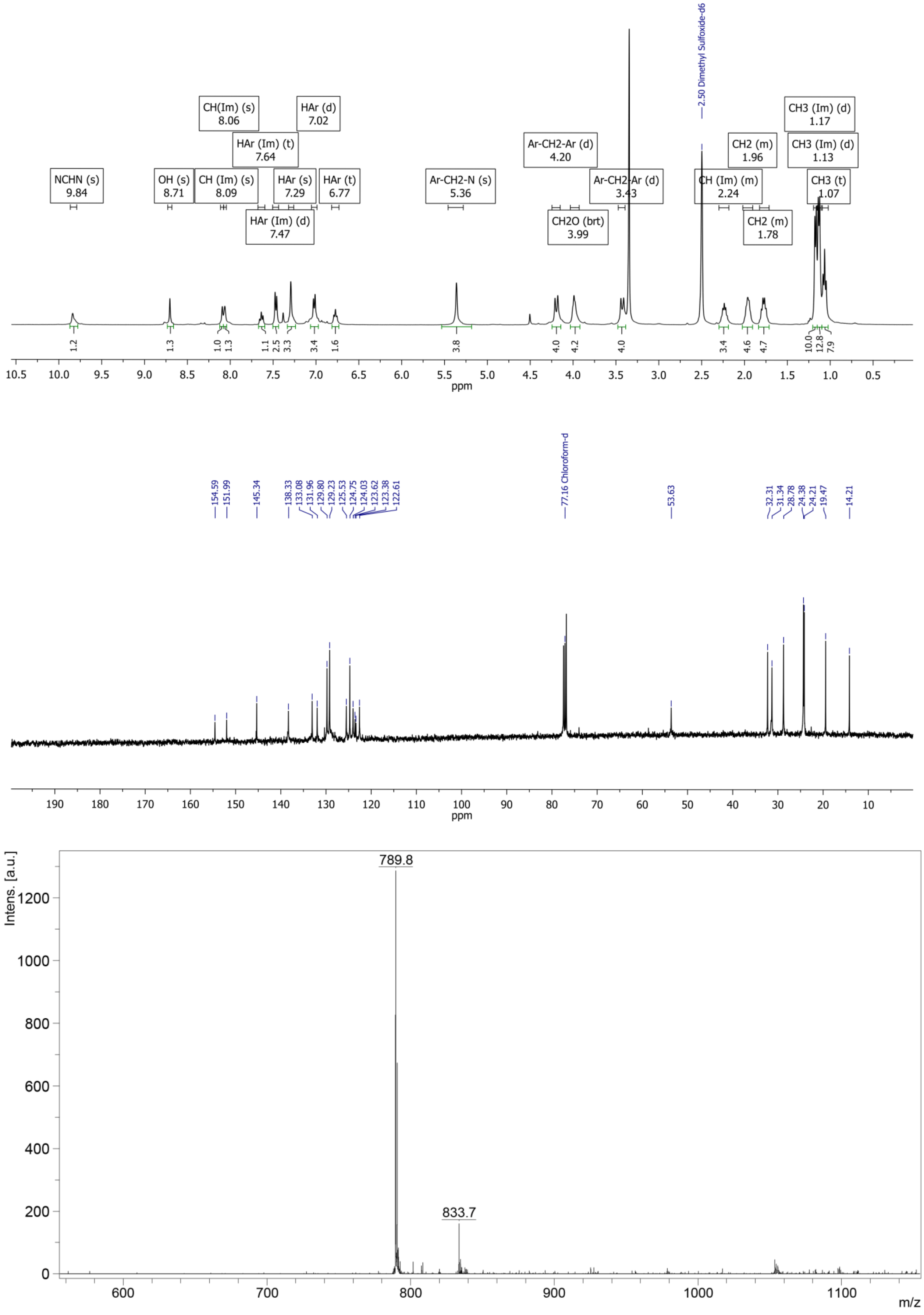

Fig. S4 NMR ^{1}H, ^{13}C and MALDI-TOF spectra of 11,23-bis[(3-(2,6-diisopropylphenyl)-1H-imidazolium-1-yl)methyl]-25,27-dihydroxy-26,28-dibutoxycalix[4]arene dichloride (**7**)

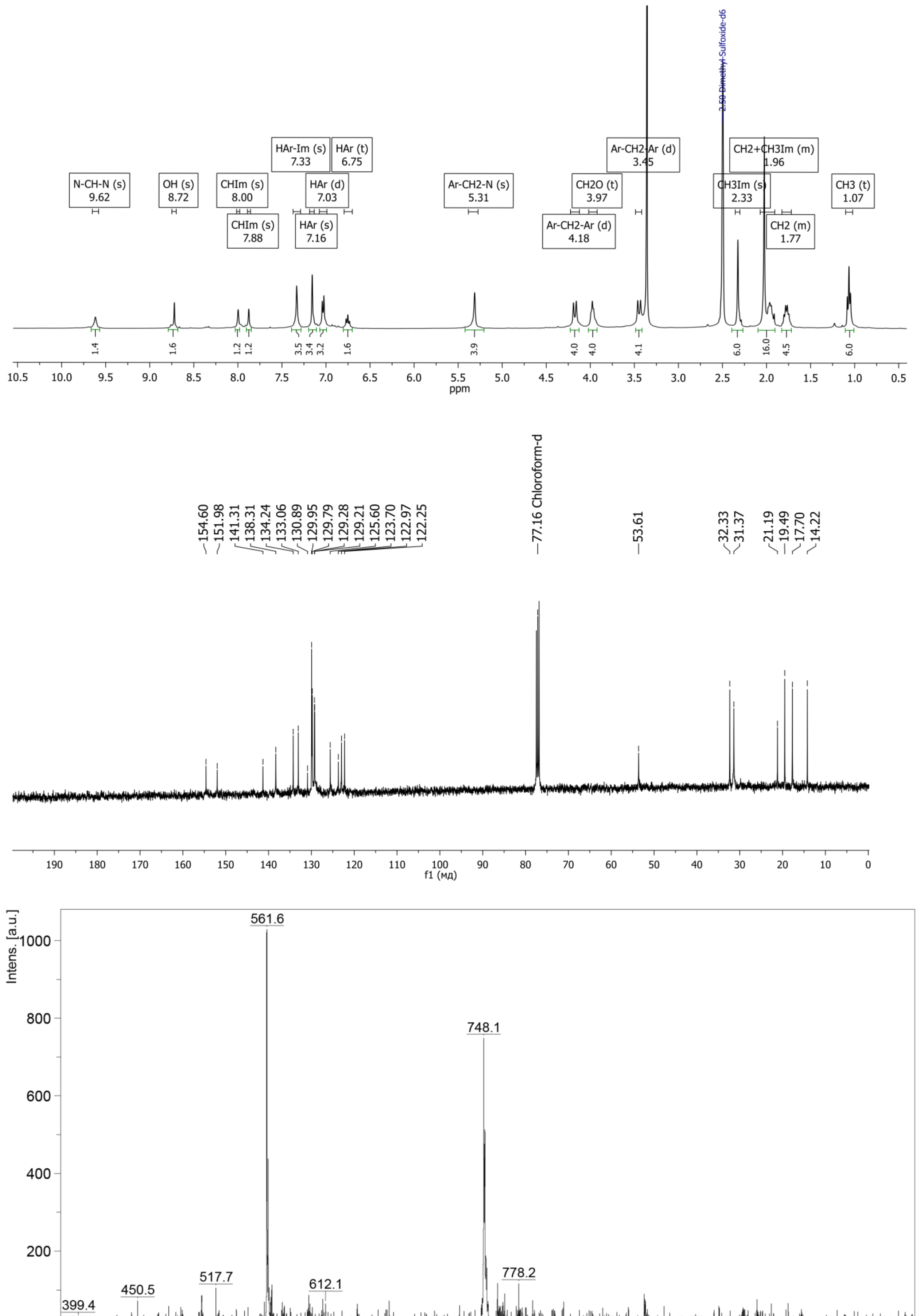

Fig. S5 NMR ^{1}H, ^{13}C and MALDI-TOF spectra of 11,23-bis[(3-(mesityl)-1H-imidazolium-1-yl)methyl]-25,27-dihydroxy-26,28-dibutoxycalix[4]arene dichloride (**8**)

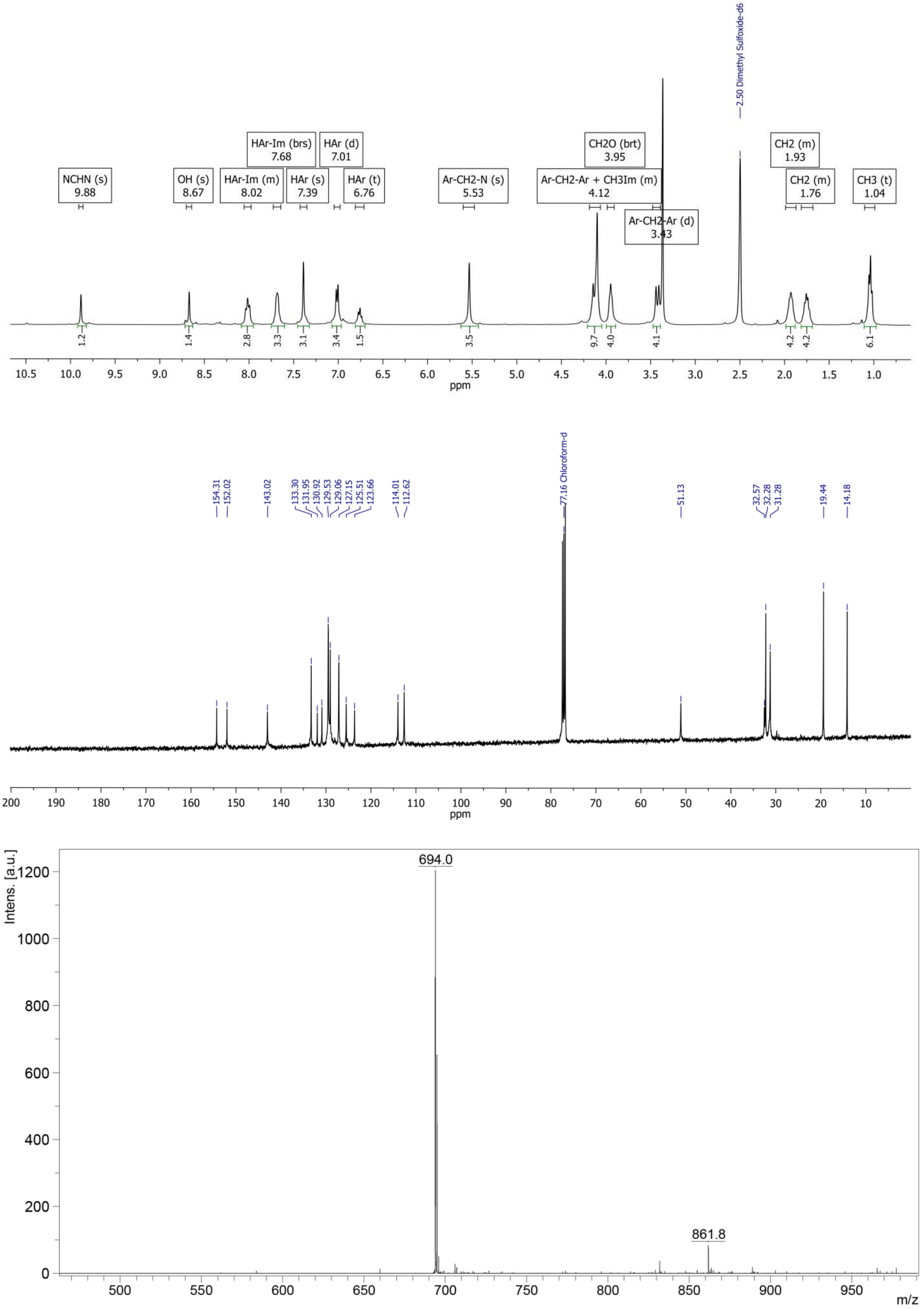

Fig. S6 NMR ^{1}H, ^{13}C and MALDI-TOF spectra of 11,23-bis[(3-methyl-1H-benzimidazolium-1-yl)methyl]-25,27-dihydroxy-26,28-dibutoxycalix[4]arene dichloride (**9**)

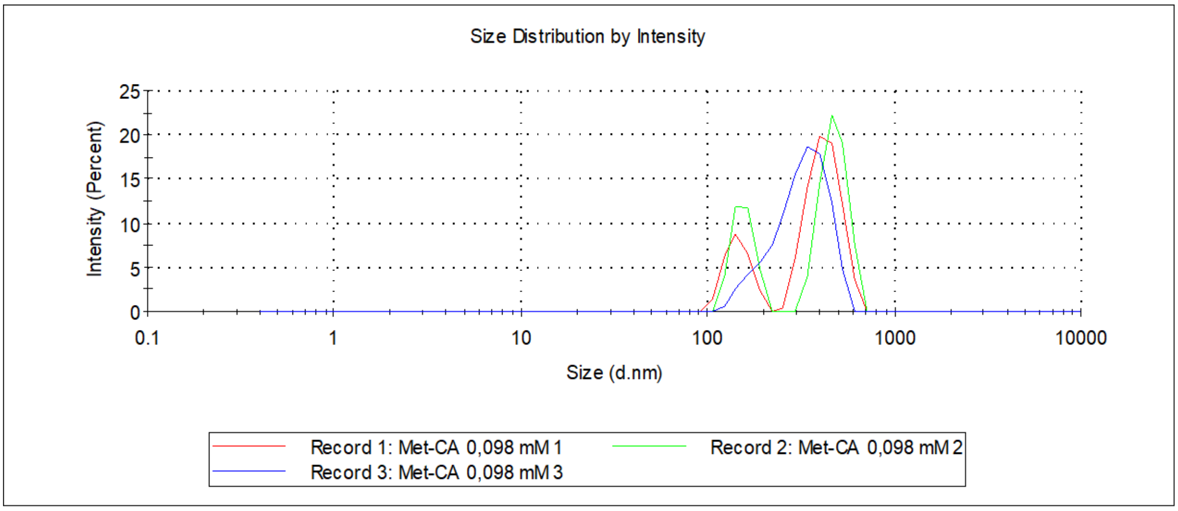
Size Distribution by Intensity
Intensity (Percent)
25
20
15
10
5
0
0.1
1
10
100
1000
10000
Size (d.nm)
Record 1: Met-CA 0,098 mM 1
Record 2: Met-CA 0,098 mM 2
Record 3: Met-CA 0,098 mM 3

(a)

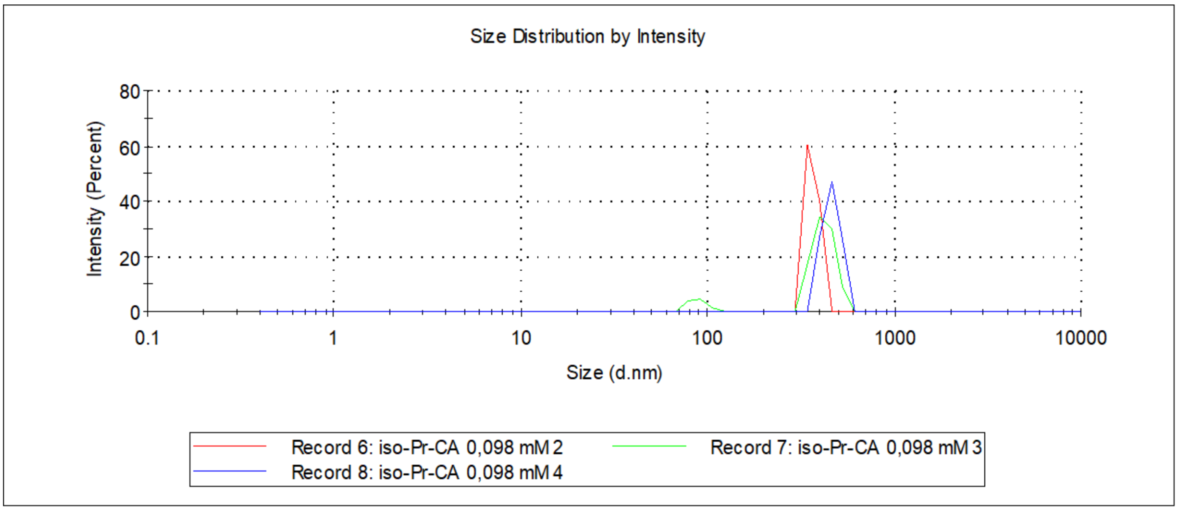
Size Distribution by Intensity
Intensity (Percent)
80
60
40
20
0
0.1
1
10
100
1000
10000
Size (d.nm)
Record 6: iso-Pr-CA 0,098 mM 2
Record 7: iso-Pr-CA 0,098 mM 3
Record 8: iso-Pr-CA 0,098 mM 4

(b)

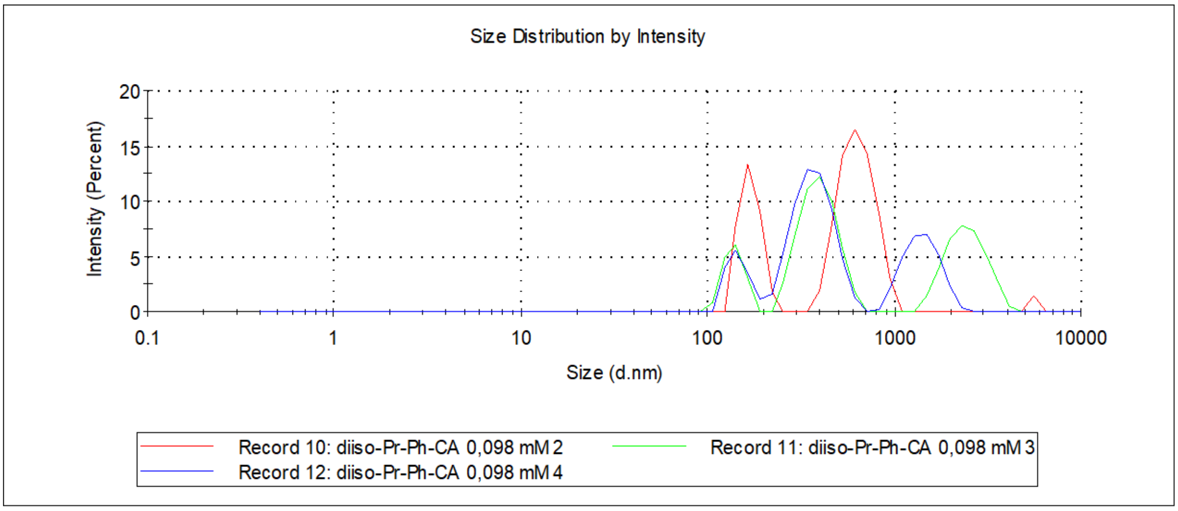
Size Distribution by Intensity
Intensity (Percent)
20
15
10
5
0
0.1
1
10
100
1000
10000
Size (d.nm)
Record 10: diiso-Pr-Ph-CA 0,098 mM 2
Record 11: diiso-Pr-Ph-CA 0,098 mM 3
Record 12: diiso-Pr-Ph-CA 0,098 mM 4

(c)

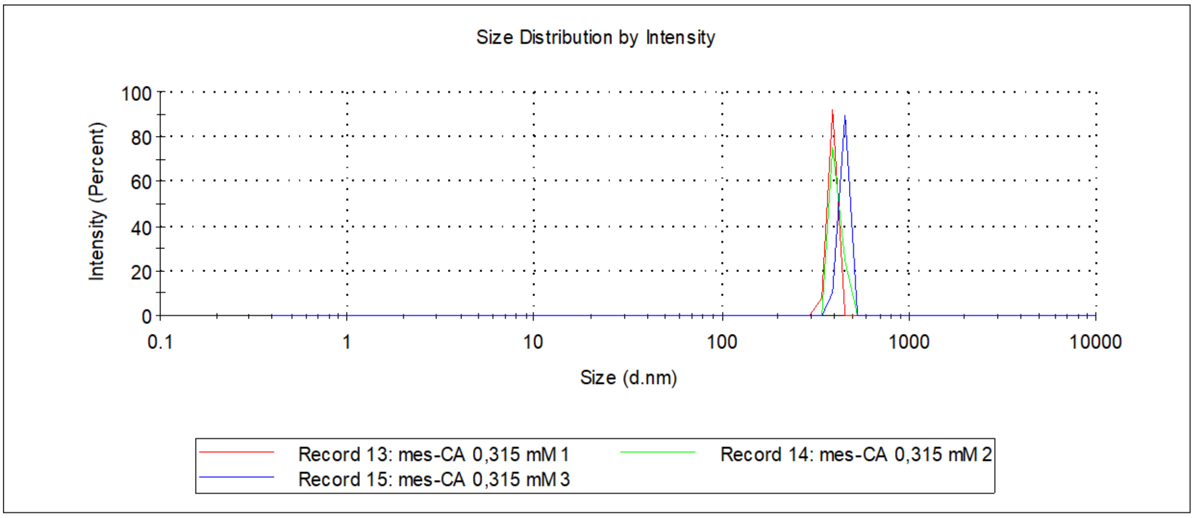

(d)

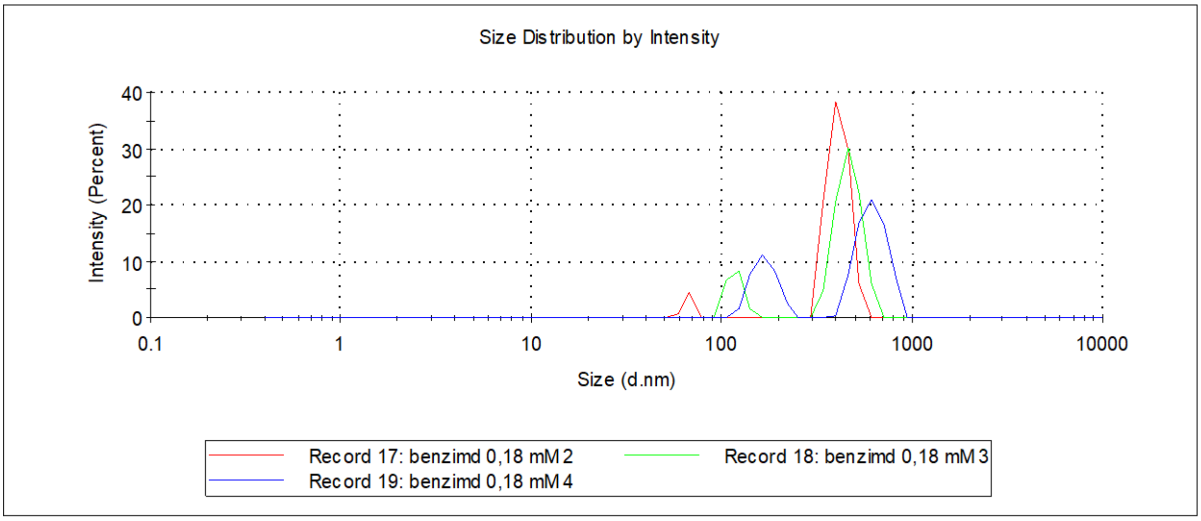

(e)

Fig. S7**.** DLS size graph of aggregates formed by **5**(a), **6**(b), **7**(c), **8**(d), **9**(e), C(**5-9**) = 97, 33, 60, 80, 180 µM.

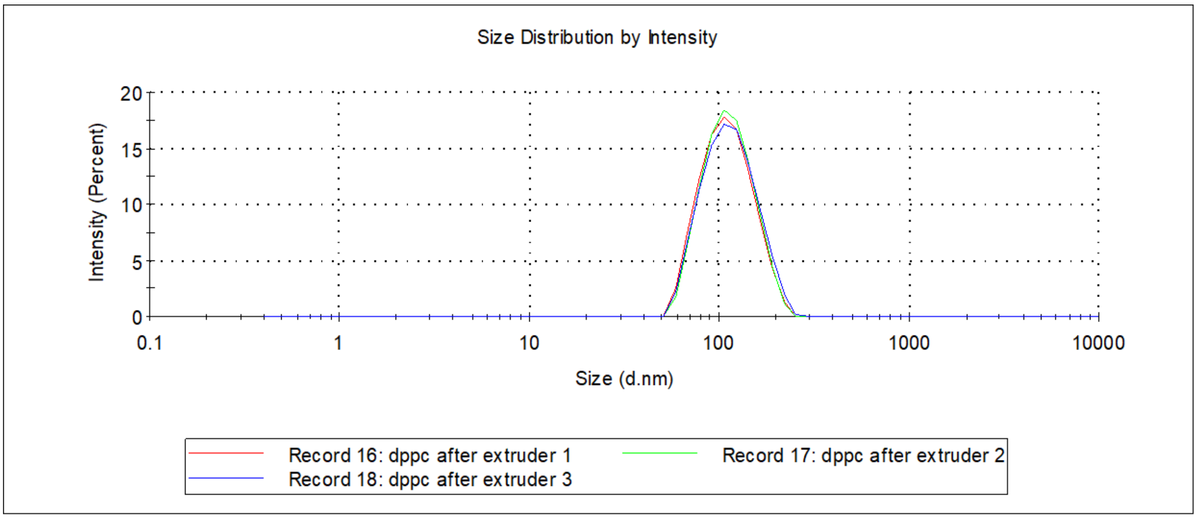
Size Distribution by Intensity
Intensity (Percent)
20
15
10
5
0
0.1
1
10
100
1000
10000
Size (d.nm)
Record 16: dppc after extruder 1
Record 17: dppc after extruder 2
Record 18: dppc after extruder 3

(a)

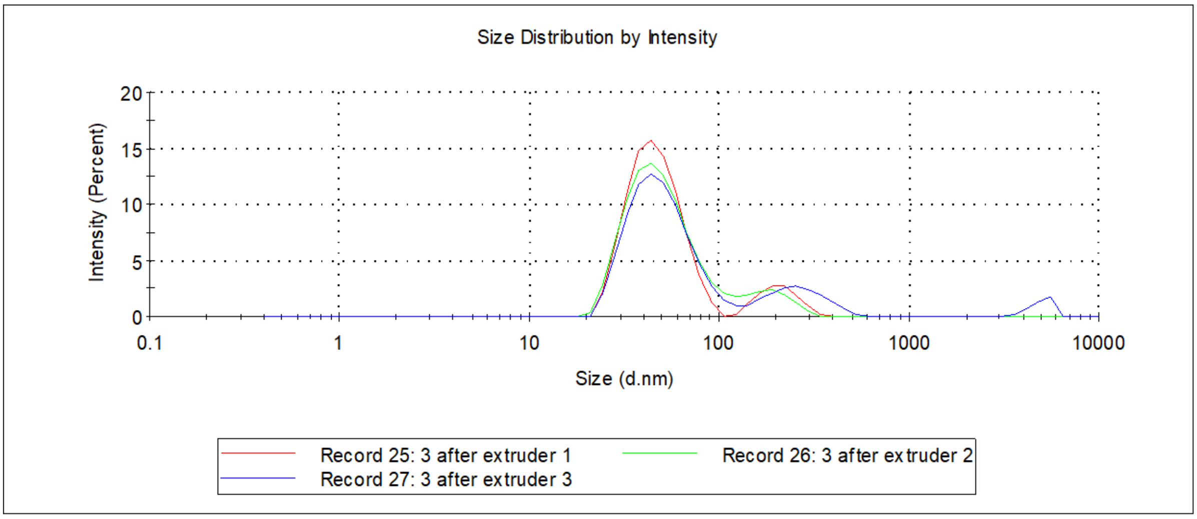
Size Distribution by Intensity
Intensity (Percent)
20
15
10
5
0
0.1
1
10
100
1000
10000
Size (d.nm)
Record 25: 3 after extruder 1
Record 26: 3 after extruder 2
Record 27: 3 after extruder 3

(b)

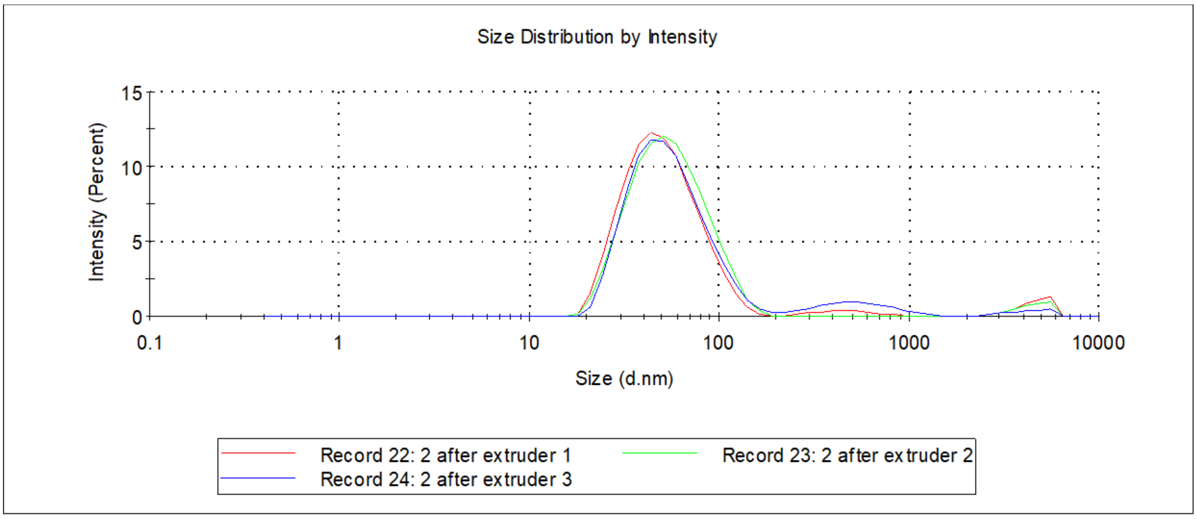
Size Distribution by Intensity
Intensity (Percent)
15
10
5
0
0.1
1
10
100
1000
10000
Size (d.nm)
Record 22: 2 after extruder 1
Record 23: 2 after extruder 2
Record 24: 2 after extruder 3

(c)

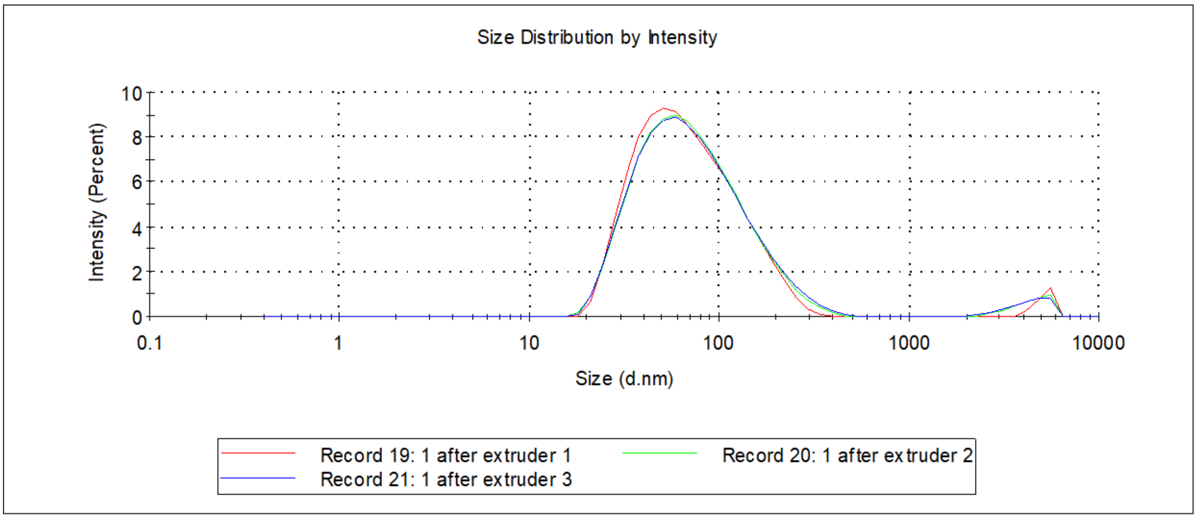

(d)

Fig. S8. DLS size graph of aggregates formed by DPPC (a) or DPPC mixed with **5,** C(DPPC) = 1 mM, C (**5**) = 0.043(b), 0.65(c), 0.98(d) mM.

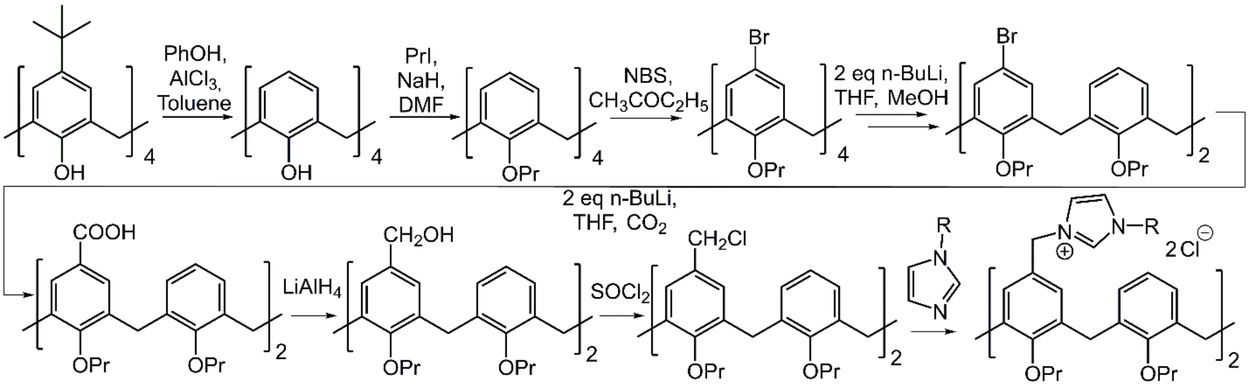

Scheme S1. Known synthetic pathway for NHC-precursors made by Schatz group.

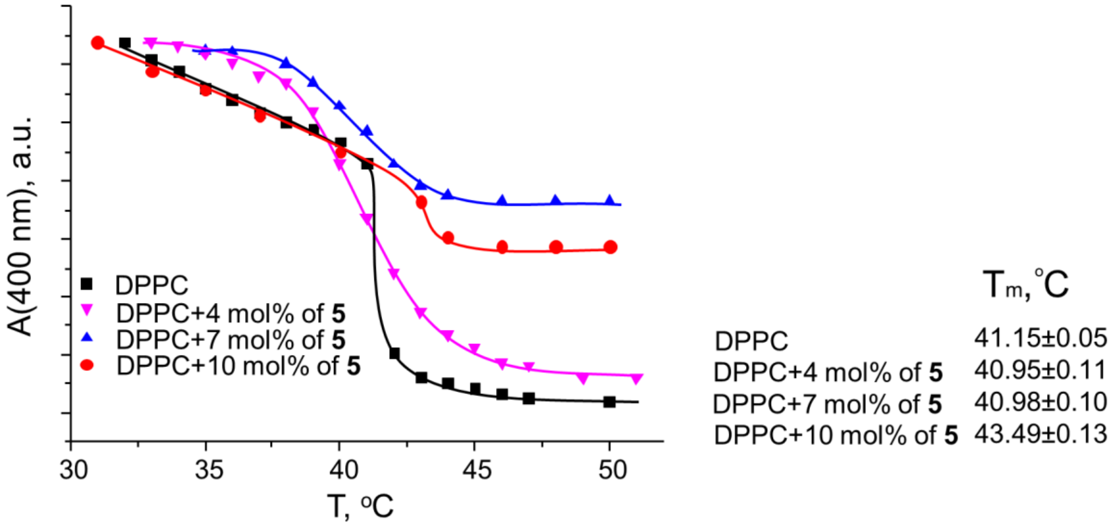

Fig. S9. T_m plots of DPPC and DPPC-**5** vesicles, C(DPPC) = 1 mM; C (**5**) = 43, 65 and 98 µM for DPPC+ 4% (**5**), DPPC+ 7% (**5**) and DPPC+ 10% (**5**), respectively; H_2O, 26-50 °C.
